# Correlation of Tinnitus Loudness and Onset Duration with Audiological Profile Indicating Variation in Prognosis

**DOI:** 10.1155/2013/205714

**Published:** 2013-09-02

**Authors:** Sunita Gudwani, Sanjay K. Munjal, Naresh K. Panda, Roshan K. Verma

**Affiliations:** ^1^Department of Otolaryngology, Postgraduate Institute of Medical Education and Research, Chandigarh, India; ^2^Speech and Hearing Unit, Department of Otolaryngology, PGIMER, Chandigarh 160012, India

## Abstract

*Purpose*. Subjective tinnitus has different forms and degrees of severity. Many studies in the literature have assessed psychoacoustic characteristics of tinnitus but hardly any of them had focused on the association of audiological profile with onset duration and loudness perception. The *aim* of this study was to evaluate existence of any association between tinnitus loudness/onset duration and audiological profile to explain differences in prognosis. *Method*. Study design was prospective. The sample consisted of 26 subjects having tinnitus, which was divided into tinnitus and nontinnitus ears. Audiological profile included pure-tone audiometry, speech audiometry, tympanometry, acoustic reflex test, and auditory evoked potentials (early and middle latency). Unpaired *t*-test was applied to compare two subgroups. Correlation and association between tinnitus onset duration/loudness perception and audiological profile were also assessed by calculating Spearman's coefficient and Fischer exact value. *Results*. The two subgroups had significant differences for pure-tone and speech audiometry hearing thresholds. A significant association was observed between the high frequency/extended high frequency and tinnitus loudness/onset duration. *Conclusion*. The changes in hearing thresholds and auditory pathway are associated with an increase in tinnitus loudness and its onset duration. This knowledge would be helpful to differentiate between severity and chronicity of the patients for planning therapeutic management and predicting prognosis.

## 1. Introduction

Subjective tinnitus is the perception of sounds by the patient without any physical presence of acoustic stimulus [1]. The perceived localization of tinnitus is reported as from one ear, from both the ears with the same or different intensity, or from inside the head [2]. It might be perceived as a weak pure tone, ringing of bells, shrill birds' chirping, noise of whizzing air, or loud noise of a jet engine. Subjective tinnitus has different forms and degrees of severity, and the diagnosis has to be made solely on the information provided by the patient [3, 4]. The severity is classified by the patient's own estimate as slight, moderate, and severe depending on the problem and annoyance faced [5]. A particular treatment that helps one patient may fail for others, suggesting that there are different forms of tinnitus which differ in their pathophysiology and their response to specific treatments [6]. The clinical subtypisation of different forms of tinnitus is an important step towards the goal of individualized promising treatment [6].

Subjective ratings of tinnitus loudness, using visual analogue scales, have been found to correlate with distress [7], although little correlation between tinnitus loudness and the impact of tinnitus on daily life was reported [8]. The usefulness of tinnitus loudness was questioned by Andersson [9], but it was stated that future research on tinnitus should focus on differences between patients with high and low annoyance [10]. Psychological treatment for tinnitus was reported as effective for loudness perception, negative affect and sleep where the improvement in loudness perception was small which disappeared at follow-up [11].

Many aspects of tinnitus are yet to be answered completely as what are the differences in tinnitus ear and nontinnitus ear; how the loudness perceived is relevant to treatment; whether it is associated with auditory changes; whether the onset was recent or long standing; and how these differences affect the prognosis of tinnitus. It was observed in our tinnitus therapy clinics that these differences seemed to play some role in prognosis and this information might help in planning focused and effective management of the subjects with tinnitus. Therefore the objective of the present study was to find any possible correlation of the perceived loudness and onset duration of tinnitus with audiological profile and its role in treatment. The null hypothesis was made of no correlation between the variables and had no impact on treatment plan.

## 2. Material and Methods

This was a nonrandomized study which included 30 subjects who were seeking treatment for their tinnitus problem in outdoor patient services of the institute. A prior approval of the institute ethics committee was obtained before commencement of the study. Due to the time constraints of the study, a small sample was selected and consisted of subjects of either sex from rural and urban backgrounds. There was a dropout of 4 subjects; hence analysis was done with 26 subjects. All the subjects had chief complaint of idiopathic subjective tinnitus with or without hearing loss. The inclusion criteria were healthy external or middle ear on clinical examination, consistent tinnitus of more than 10-week duration, and those who gave their written consent for the study. Patients with external or middle ear pathology, suspected Meniere's disease or otosclerosis, history of ototoxicity, sudden hearing loss, or ear trauma or having any systemic disorders were excluded.

The cases were subjected to a complete clinical and audiological assessment. The audiological assessment consisted of pure-tone audiometry (conventional audiometry), high frequency audiometry (HFA), extended high frequency audiometry (EHA), speech audiometry (speech reception threshold (SRT), speech discrimination score (SDS), most comfortable level (MCL), and uncomfortable level (UCL)), Tympanometry, acoustic reflex testing (ART) (ipsi and contra, reflex decay), auditory brainstem responses (ABR), middle latency responses (MLR), tinnitus matching (pitch and loudness), and residual inhibition (RI). Otoacoustic emission (OAEs) and magnetic resonance imaging (MRI) were done in few selected cases. The different equipment used for the audiological investigations included Madsen orbiter 922 clinical audiometer, Siemens SD 30 tympanometer, evoked potential system developed by Intelligent Hearing System, USA, and otoacoustic system developed by Intelligence Hearing System, USA. The assessment also included subjective scaling (5-point scale) of annoyance and sleep disturbance due to tinnitus. These investigations were done in two visits of 50–60 minutes each prior to start of treatment. The management was planned as combination of masking therapy, environment enrichment with music, and cognitive behavior therapy.

*Statistical Analysis.* The data was subjected to the values of mean and median for central tendency and standard deviation (SD) for variability. Unpaired *t*-test was applied for comparison of tinnitus and nontinnitus ears. Spearman's correlation coefficient (rho) was calculated between psychoacoustic characteristics and the audiological profile of tinnitus ears. Fischer's exact test was used as a nonparametric inferential test to verify any association between tinnitus onset duration and type of hearing loss. Similarly it was also used to verify any association of tinnitus loudness with the type of hearing loss. All significance tests were two tailed and conducted at or above the 95% significance level (*P* < 0.05).

## 3. Observations and Results

The 26 subjects included in the study ranged from 16 to 45 years of age with a mean 37.12 years ± SD of 8.57. Males and females were equally distributed. 

## 4. Psychoacoustic Characteristics of Tinnitus

The psychoacoustic characteristics included tinnitus ear, duration of tinnitus since onset, pitch matching, loudness matching, and residual inhibition of tinnitus. 28% of the subjects had tinnitus in the right ear, 40% had in the left ear, and 32% had tinnitus perception binaurally. The sample was divided into two groups, tinnitus ears (35 ears) and nontinnitus ears (17 ears).

The majority of tinnitus ears (72%) had onset duration longer than six months. 22.9% of the ears had duration of tinnitus perception <0.5 yrs (2.5 months–6 months), 25.7% of the ears had tinnitus perception since 0.5 to 1 year, 2.9% since 1.0 to 1.5 years, 14.3% since 1.5 to 2 years, 17.1% since 2.0 to 5.0 years, and 17.1% since >5 years. 

Tinnitus matching was done where 44% of the ears had pitch perception <6000 Hz and 48% had perception ≥6000 Hz. Loudness matching showed that 24% of the subjects had faint tinnitus perception ≤30 dB HL, 44% had loud (31 to 50 dB HL), 20% had too loud (51 to 70 dB HL), and 12% had extremely loud level (71 to 90 dB HL). Loudness matching was done contralaterally in hearing level (HL) units rather than in sensation level units (SL) [12] as the minimum masking level was measured ipsilaterally in SL [11]. Residual inhibition was considered positive when after masking there was a decrease in perceived loudness by ≥5–7 dB or change in pitch >100 Hz and he/she reported subjective decrease in loudness and/or pitch change. It was found positive in 68% of the subjects. The rest of the 32% of the subjects did not show residual inhibition.

## 5. Subjective Rating

The level of annoyance was subjectively reported by the individuals on a 5-point scale, where 0 = no annoyance, 1 = little annoyance, 2 = average annoyance, 3 = high annoyance, and 4 = intolerable. All of the subjects reported level of annoyance as average or more than average, that is, ≥2. The sleep disturbance due to tinnitus was rated on a 5-point scale as 0 = never affected, 1 = rarely affected, 2 = sometimes affected, 3 = mostly affected, and 4 = always affected. Out of the total 26 subjects, 22 reported sleep disturbance as sometimes affected due to tinnitus, two of the patients reported as mostly affected (since <6 weeks), and one reported as always affected (since 2 weeks).

## 6. Pure-Tone Audiometry

Based on pure-tone audiometric thresholds, PTA1 (average of 500, 1000, and 2000 Hz) was calculated for both right and left ears (conventional audiometry), and hearing status of the subjects was categorized into mild, moderate, moderate to severe, severe, and profound (WHO Classification, 1980; ISO-R.389: 1970) [13]. Similarly the average of 4000, 8000, and 10000 Hz was calculated as PTA2 (high frequency audiometry) and the average of 12000, 14000, and 16000 Hz as PTA3 (extended high frequency audiometry) for both ears. 

**Figure 1:**
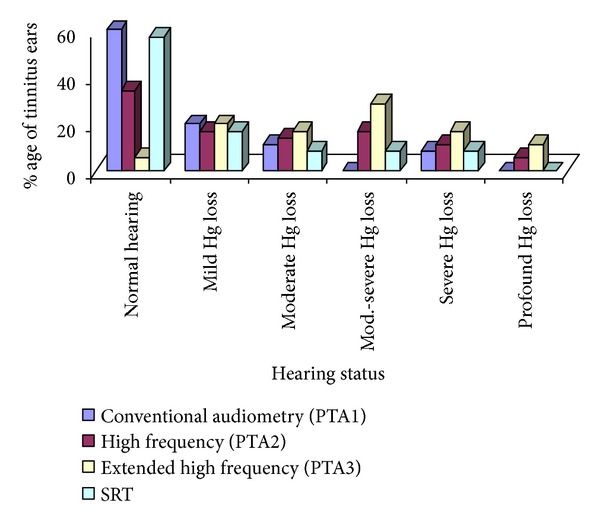
Hearing status of tinnitus ears.

**Figure 2:**
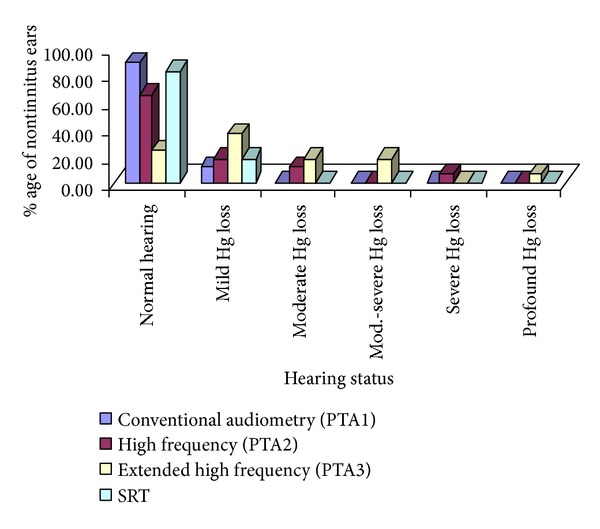
Hearing status of nontinnitus ears.

The data shows that 60% (21 ears) of tinnitus ears had normal hearing on conventional audiometry (PTA1), 20% (7) ears had mild hearing loss, 11.4% (4) had moderate hearing loss, and 8.6% (3) of tinnitus ears had severe hearing loss. None of the tinnitus ears had profound hearing loss (Figure 1). The data of nontinnitus ears show that on conventional audiometry (PTA1), 88.2% (15) ears had normal hearing and 11.8% (2) ears had mild hearing loss (Figure 2).

On high frequency audiometry (PTA2), 34.3% (12 ears) of the tinnitus ears had normal hearing, 17.1% (6 ears) had mild hearing loss, 14.3% (5) ears had moderate hearing loss, 17.1% (6) ears had moderate to severe hearing loss, 11.4% (4) ears had severe hearing loss, and 5.7% (2) of the tinnitus ears had profound hearing loss (Figure 1). For nontinnitus ears on PTA2, 64.7% (11) had normal hearing, 17.6% (3) ears had mild hearing loss, 11.8% (2) ears had moderate hearing loss, and 5.9% (1) ears had severe hearing loss (Figure 2).

According to extended high frequency audiometry (PTA3), 5.7% (2 ears) of the tinnitus ears had normal hearing, 20% (7) ears had mild hearing loss, 17.1% (6) ears had moderate hearing loss, 28.6% (10) ears had moderate to severe hearing loss, 17.1% (6) ears had severe hearing loss, and 11.4% (4) of the tinnitus ears had profound hearing loss. For nontinnitus ears on PTA3, 23.5% (4) had normal hearing, 35.5% (6) ears had mild hearing loss, 17.6% (3) ears had moderate hearing loss, 17.6% (3) ears had moderate to severe hearing loss, and 5.8% (1) ear had profound hearing loss (Figure 2). 

## 7. Speech Audiometry

The speech reception threshold (SRT) was normal, that is, ≤25 dBHL in 57.1% (20 ears) of the tinnitus ears, and 17.1% (6) of the tinnitus ears had SRT between 26 and 40 dBHL. It shows that the majority (74.2%) of tinnitus ears had good speech reception at general conversational levels (Figure 1). Speech discrimination score (SDS) was good (≥90%) in the majority of the tinnitus ears, that is, 71.4% (25 ears). SDS was found between 80 and 90% in 20% (7) of the tinnitus ears. That means that 91.4% of the tinnitus ears had good discrimination of speech. Only 8.6% (3) of tinnitus ears had SDS below 80%. Uncomfortable level (UCL) was observed to be normal (≥100 dBHL) in 80% of the tinnitus ears while the rest 20% had UCL 90–100 dBHL. Otoacoustic emissions (OAEs) were done for these 20% subjects to ensure the outer hair cells' (OHC) intactness. Those subjects with OHC poor functioning (absent/reduced DPOAEs) were excluded from the study. Uncomfortable level (UCL) was ≥90 dBHL in all of the tinnitus ears (100%).

The SRT was normal, that is, ≤25 dBHL in 82.3% (14ears) of the nontinnitus ears, and the rest 17.64% (3) of the ears had SRT between 26 and 40 dBHL (Figure 2). Speech discrimination score SDS was good (≥90%) in all of the nontinnitus ears. UCL was observed normal (≥100 dBHL) in 85% of the non-tinnitus ears, while the rest 15% had UCL 90–100 dBHL. Otoacoustic emissions (OAEs) were done for these 15% subjects to ensure the outer hair cells' (OHC) intactness. All of the non-tinnitus ears (100%) had UCL ≥90 dBHL.

## 8. Auditory Brainstem Evoked Responses (ABR)

The interpeak latency (IPL) of wave I-III was considered normal as 1.6–2.4 ms, shortened as <1.6 ms, and prolonged as >2.4 ms. Wave III-V IPL was normal as 1.8–2.2 ms, shortened as <1.8 ms, and prolonged as >2.2 ms. The IPL of wave I–V was normal as 3.6–4.4 ms, shortened as <3.6 ms, and prolonged as >4.4 ms. The correction (0.1 ms for every 10 dB of hearing loss above 50 dB) was applied to calculate the absolute latency of wave V when the subject had hearing loss greater than 50 dBHL at 4000 Hz [14].

The IPL of wave I–III was observed as normal in 82.8% (29 ears) of the tinnitus ears, prolonged in 8.6% (3) ears, and no response in 8.6% (3) ears. None of the ears had shortened wave I–III interpeak latency. In non-tinnitus interpeak latency of wave I–III was normal in 94.1% of the ears (16 ears), and prolonged in 5.9% (1) of the ears. None of the ears had shortened IPL of wave I–III. 

Wave III–V interpeak latency was normal in 17 tinnitus ears (48.6%), shortened in 16 ears (45.7%), and prolonged in one tinnitus ear (2.8%) and there was no response in one ear (2.8%). In non-tinnitus ears wave III–V was observed as normal in 10 ears (58.8%) and shortened in seven ears (41.2%). 

Data shows IPL of wave I–V as normal in 30 tinnitus ears (85.7%), shortened in one ear (2.8%), prolonged in one ear (2.8%), and no response in three ears (8.6%). IPL of wave I–V in non-tinnitus was normal in 88.2% (15) of the ears, shortened in 5.9% (1), ears and prolonged in 5.9% (1) of the ears. MRI was normal in this one subject. Magnetic resonance imaging (MRI) was recommended in subjects with unexplained prolonged IPL of waves to ensure no retrocochlear pathology. Those with retrocochlear pathology were excluded from the study.

## 9. Middle Latency Evoked Responses (MLR)

Peaks observed during MLR were Na, Pa and Nb waves, out of these amplitude of waves Na and Pa were analyzed. The amplitude of waves Na and Pa was considered normal as ≥0.50 *μ*V and abnormal as <0.50 *μ*V.

Wave Na had normal amplitude in 94.3% (33 ears) of the tinnitus ears, abnormally low in 2.8% (1) of the ears, and no response in 2.8% (1) of the tinnitus ears. Amplitude of wave Pa was normal in 32 ears (91.4%), abnormal in 2 ears (5.7%), and of no response in 1 tinnitus ear (2.8%). In non-tinnitus the amplitude of waves Na and Pa was observed to be as normal in all of the 17 ears (100%).

## 10. Comparison between Tinnitus and Nontinnitus Ears (Table 1)

The two groups tinnitus and non-tinnitus ears were compared with unpaired “*t*”-test. The comparison was made between all the measured audiological parameters, but the results of statistically significant findings are depicted in the tables. Table 1 shows that the two groups had significant differences for pure-tone audiometry and speech audiometry. For ABR and MLR measurements, only absolute latency of wave V was significantly different between the two groups, and all of the rest parameters were found nonsignificant.

## 11. Correlation between Onset Duration of Tinnitus and Audiological Profile (Table 2)

Positive correlation was observed between tinnitus onset duration and high frequency thresholds (4000, 8000, and 10000 Hz and high frequency average PTA2). Similarly positive correlation was also observed for extended high frequency threshold (12000 and 14000 and EHF average PTA3). Positive correlation was also observed between tinnitus duration and absolute latencies of waves III and V of ABR (Table 2).

Negative correlation was observed between duration and speech discrimination score (SDS) in tinnitus ears. None of the correlation coefficient values was statistically significant between tinnitus duration and interpeak latencies of ABR waves. Similarly, none of the correlation coefficient values was statistically significant between duration and amplitude of middle latency evoked response (MLR) waves Na and Pa.

## 12. Correlation of Tinnitus Loudness with Audiological Profile (Table 3)

Significant correlation was observed between perceived tinnitus loudness and conventional audiometric thresholds and average. Similarly significant correlation was found for high frequency and extended high frequency thresholds. As shown in Table 3, tinnitus loudness was also significantly correlated with speech reception threshold (SRT), speech discrimination score (SDS), and most comfortable level (MCL). None of the ABR and MLR parameters were significantly correlated with tinnitus loudness.

## 13. Association between Tinnitus Onset Duration and Audiological Profile (Table 4)

Fischer's exact test was used to evaluate the association between tinnitus onset duration and type of hearing loss. Statistically significant association value (*f* = 21.149, *P* < 0.05) was found between duration and conventional hearing loss (PTA1). Similarly significant value (*f* = 29.98, *P* < 0.05) between duration and high frequency hearing loss (PTA2) was observed. Association of duration with extended high frequency hearing loss (PTA3) (*f* = 24.25) was nonsignificant.

## 14. Association between Tinnitus Loudness and Audiological Profile (Table 4)

Fischer's exact test was also used to assess association of perceived tinnitus loudness with type of hearing loss. Association was significant between loudness and conventional hearing loss (PTA1) (*f* = 19.544; *P* < 0.01). Association was also statistically significant (*f* = 21.730; *P* < 0.05) between tinnitus loudness and high frequency hearing loss. It was nonsignificant for extended high frequency hearing loss (*f* = 18.507).

## 15. Therapeutic Variations

To control the variability, therapy was given by one clinician to all subjects. Combination management of masking therapy, environment enrichment (with music before sleep to shift attention from tinnitus/interference) and individual cognitive behavior therapy (CBT) was given for two weeks. Improvement was defined objectively as change in loudness level ≥15 dBHL plus subjectively as improvement on a 5-point scale of annoyance rating and a 5-point scale of sleep disturbance. When the loudness level was faint or loud and onset duration was <0.5 years, the prognosis reported by the subjects was good immediately after completing therapy and six months later. Given similar management the subjects with loudness perception of 51 dB HL to 70 db HL (too loud) and/or onset duration 0.5–1 year reported fair improvement immediately after the therapy and some relapse by 2-3 months hence needed further therapeutic management. When onset duration of tinnitus was longer than 2 years and/or loudness was 71−90 dbHL (extremely loud) with this therapeutic plan, there was hardly any improvement and the subjects were shifted to other management strategies after 2 weeks like combination with electrical stimulation.

## 16. Discussion

In the study, the majority (60% of tinnitus ears and 80% of non-tinnitus ears) of the subjects had normal hearing on conventional pure-tone average (PTA1) which goes in accordance with the results published by Roberts et al. [15]. Other studies have reported contradictory findings showing an increased prevalence of hearing loss with tinnitus perception [16]. Reason might be because of the frequencies tested that were not divided into different regions of the spectrum as has been done in our study. However, comparison of tinnitus and non-tinnitus ears showed that hearing thresholds at 1000 Hz, 2000 Hz, PTA1, SRT, and SDS had significant differences explaining the increased prevalence of hearing loss with tinnitus, as observed in previous studies [16].

Hearing thresholds were poorer in high frequency region 4000, 8000, and 12000 Hz. Roberts et al. [15] also reported similar findings that tinnitus subjects had high frequency hearing loss. In the present study, these thresholds were higher when duration since onset was longer or when perception of tinnitus was louder. This suggests that tinnitus is associated with changes in the auditory system as the duration and loudness increase or viceversa.

Most of the tinnitus subjects in our study had hearing loss in both ears by extended high frequency (EHF) average (94.2% of tinnitus ears and 76.5% of non-tinnitus ears). However, on comparison of tinnitus and non-tinnitus ears, significant differences were observed for extended high frequency hearing thresholds and PTA3. Barnea et al. [17] found that extended high frequency hearing thresholds in tinnitus and non-tinnitus subjects were not significantly different. The disagreement with the present study might be the difference of tinnitus duration, as observed by positive correlation between duration and EHF thresholds, that is, longer the tinnitus onset duration higher was the extended high frequency thresholds or EHF average (PTA3). It was also found with correlation based on tinnitus loudness that louder tinnitus was associated with higher thresholds at EHFs. The findings of the present study disagree with the previous study [8] concluding little correlation between tinnitus loudness and the impact of tinnitus on daily life as measured by tinnitus handicap inventory. The differences might be due to correlating tinnitus loudness with the degree of hearing loss in the present study instead of the handicap in daily living. The positive correlations of the present study indicate that duration and loudness of tinnitus are associated with high frequency and extended high frequency processing sites of the auditory system.

ABR studies in the literature do not reveal any shortening of IPL I–III, III–V, or I–V waves. However, we found wave III–V interpeak latency shortening (<1.8 msec) in many subjects (45.7% in tinnitus ears and 41.2% in non-tinnitus ears). This indicates lesser conduction time of auditory stimulus at higher brainstem level. Moller [18] studied the compound action potential and brainstem evoked potentials from exposed eighth nerve in patients with intractable tinnitus. They reported that absolute latency of wave III was unchanged but latency of wave V was significantly shorter due to hyperactivity of some structures in ascending auditory pathway [18]. The shortening of III–V IPL observed in both tinnitus and non-tinnitus ears in the present study can be explained by crossover of neural network leading to binaural representation at high brainstem level. Positive correlation was seen between tinnitus duration and absolute latency of waves III and V, indicating that, as the onset duration increases, ABR latencies get worse and this worsening is not due to poorer hearing threshold as latency correction was applied according to Selters and Brackmann (1977) formula [14]. No significant correlation was observed for MLR waves Na and Pa amplitude in the present study although a previous study by Gerken et al. [19] reported that there is a selective alteration of MLR generators in different forms of tinnitus.

It therefore becomes clear that tinnitus perception is associated with changes in auditory pathway especially the areas responsible for high frequencies and extended high frequencies processing. And these changes are correlated with prolongation of time and increase in intensity of tinnitus perception.

According to psychological models, promising treatment for tinnitus is to modify the central nervous substrate of tinnitus and consequently its percept [20]. Tyler [21] reported that the psychological factors to be considered are habituation, learning, attention (failure to shift away attention from tinnitus), and cognitive aspects (nonadaptive and less functional ways of thinking about tinnitus). One previous study added the use of a noisegenerator to a 10-session CBT group treatment, although the noise generator was found helpful for patients with a co-occurrence of hyperacusis [7]. The same was done in the present study by giving combination of masking and CBT. It was observed that when loudness and/or onset duration was higher, the improvement was either slow, less, or required an other combination of therapeutic strategies. Thus, this indicates that onset duration and loudness of tinnitus are important aspects in planning treatment.

This study has some limitations which could be addressed in future studies. Though a thorough case history of the subjects was taken, the possibility of pretinnitus hearing loss or audiological problems cannot be fully ruled out. Inclusion of radiological investigations particularly functional MRI would have been beneficial and would have added to the validity of the changes. The bigger sample would be safe to generalize the findings.

## 17. Conclusions

The duration and/or loudness of tinnitus perception has strong association with changes of auditory system. There might be progressive changes on hearing and auditory pathway due to longer onset duration/tinnitus loudness or vice versa. This information should be collected during assessment to be used for planning management in a focused and effective manner. It might also be used for predicting prognosis.

## Figures and Tables

**Table 1:** Comparison of tinnitus versus nontinnitus ears.

Variable	Tinnitus ears		Non-tinnitus ears		“*t*”-value
	Mean	S.D.	Mean	S.D.	
1000 (Hz)	26.14	20.83	17.94	5.60	2.17*
2000 (Hz)	31.86	23.36	18.82	9.10	2.88**
4000 (Hz)	38.29	23.54	20.88	15.13	3.22**
8000 (Hz)	46.43	30.55	29.41	24.68	2.15*
10000 (Hz)	44.69	27.27	28.82	23.09	2.15*
12000 (Hz)	52.97	26.30	37.35	22.92	2.16*
PTA1 (dB)	27.43	19.96	18.53	5.52	2.45*
PTA2 (dB)	44.55	26.87	26.37	19.86	2.74**
PTA3 (dB)	55.42	20.24	41.47	20.22	2.30*
SRT (dB)	30.54	20.38	18.76	6.10	3.14**
SDS (%age)	92.69	10.30	99.29	2.11	3.64**
MCL (dB)	54.57	19.18	43.47	8.26	2.91**
Wave V (ms)	5.67	0.22	5.54	0.19	2.22*

*P < 0.05; **P < 0.01. PTA: pure-tone average; SRT: speech reception threshold; SDS: speech discrimination score; MCL: most comfortable level; Wave V: ABR wave V absolute latency.

**Table 2:** Correlation between tinnitus duration and audiological profile.

Variable	Spearman's correlation coefficient (rho)
4000 (Hz)	0.517**
8000 (Hz)	0.427*
10000 (Hz)	0.376*
HF avg. (dB)	0.493*
12000 (Hz)	0.487**
14000 (Hz)	0.414*
EHF avg. (dB)	0.489**
SDS (%age)	−0.391*
MCL (dB)	0.347*
Wave III (ms)	0.391*
Wave V (ms)	0.430*

*P < 0.05; **P < 0.01. HF avg.: high frequency average (PTA2); EHF avg.: extended high frequency average (PTA3); SDS: speech discrimination score; MCL: most comfortable level; Wave III: ABR wave III absolute latency; Wave V: ABR wave V absolute latency.

**Table 3:** Correlation between tinnitus loudness and audiological profile.

Variable	Spearman's correlation coefficient (rho)
250 (Hz)	0.346*
500 (Hz)	0.385*
1000 (Hz0	0.484**
2000 (Hz)	0.519**
Con. avg. (dB)	0.529**
4000 (Hz)	0.655**
8000 (Hz)	0.632**
10000 (Hz)	0.606**
HF avg. (dB)	0.648**
12000 (Hz)	0.627**
14000 (Hz)	0.450*
EHF avg. (dB)	0.628**
SRT (dB)	0.466**
SDS (%age)	−0.616**
MCL (dB)	0.514**

*P < 0.05; **P < 0.01. Con. avg.: conventional audiometry average (PTA1); HF avg.: high frequency average (PTA2); EHF avg.: extended high frequency average (PTA3); SDS: speech discrimination score; MCL: most comfortable level.

**Table 4:** Association between audiological profile and tinnitus duration/loudness by Fischer's exact test.

Variable	Association with	Fischer's exact coefficient (f)
PTA1	Onset duration	21.15*
PTA2	Onset duration	29.98*
PTA3	Onset duration	24.25
PTA1	Tinnitus loudness	19.54**
PTA2	Tinnitus loudness	21.73*
PTA3	Tinnitus loudness	18.50

*P < 0.05; **P < 0.01. Con. avg.: conventional audiometry average (PTA1); HF avg.: high frequency average (PTA2); EHF avg.: extended high frequency average (PTA3).
